# Cancer incidence in UK electricity generation and transmission workers, 1973–2015

**DOI:** 10.1093/occmed/kqz082

**Published:** 2019-08-03

**Authors:** T M Sorahan

**Affiliations:** Institute of Applied Health Research, University of Birmingham, Edgbaston, Birmingham, UK

**Keywords:** Cancer incidence, electricity supply industry

## Abstract

**Background:**

Long-term health outcomes in cohorts of workers from the electricity supply industry have been studied.

**Aims:**

The aim of the study was to examine updated cancer incidence findings among a cohort of UK electricity generation and transmission workers.

**Methods:**

Cancer morbidity experienced by 81 616 employees of the former Central Electricity Generating Board of England and Wales was investigated for the period 1973–2015. All employees had worked for at least 6 months with some employment between 1973 and 1982. Standardized registration ratios (SRRs) were calculated based on national rates.

**Results:**

Overall cancer morbidity was slightly below expectation in males. Significant excesses were found in male workers for mesothelioma (observed [Obs] 763, SRR 326), skin cancer (non-melanoma) (Obs 5616, SRR 106), and prostate cancer (Obs 4298, SRR 106), and in female workers for cancer of the small intestine (Obs 13, SRR 220), nasal cancer (Obs 11, SRR 407), and breast cancer (Obs 758, SRR 110). More detailed analyses showed important contrasts, particularly for mesothelioma, lung cancer, skin cancer, prostate cancer and breast cancer.

**Conclusions:**

A clear occupational excess of mesothelioma was not matched by a corresponding excess of asbestos-induced lung cancer. Confident interpretation of the excesses of cancers of the nasal cavities and small intestine is not possible, although occupational exposures received in this industry may well not be involved. An excess of skin cancer in transmission workers may be associated with outdoor working.

**Key learning points**

**What is already known about this subject:**

- This survey was set up in the 1980s to learn more about the long-term health of employees in the UK electricity supply industry.
- In the intervening years, a series of 11 papers have found no convincing links between estimated exposure to magnetic fields and a number of health outcomes.
- This new report was prepared to provide a more complete assessment of cancer risks in the cohort, incorporating a further 6 years of follow-up data.

**What this study adds:**

- The cohort continues to experience an occupational excess of mesothelioma without any matching excess of lung cancer.
- Outdoor working may have been a factor in excess of skin cancer (non-melanoma).
- Excesses in females for nasal cancer and cancer of the small intestine were not matched by similar findings in males.

**What impact this may have on practice, policy or procedure:**

- The findings reinforce the need for regulations that protect workers from asbestos exposure and the advice given to outdoor workers concerning sun exposure.
- The findings provide indirect evidence that further control of magnetic fields exposure is probably not needed.
- Employees who have been exposed to asbestos should continue to be encouraged not to smoke to reduce the risk of asbestos-related lung cancer.

## Introduction

A cohort of UK electricity supply industry workers (power stations, substation or transmission sites, non-operational sites) was established in the 1980s to investigate whether such work was the cause of non-malignant lung disease. More recently, concerns that electromagnetic field (EMF) exposure may cause brain cancer, leukaemia, or have a role in neurodegenerative or cardiovascular diseases have been the focus of epidemiological studies in this industry [1–8], and a number of reviews are available [9–12]. Kheifets *et al.* [12] concluded that the literature on occupational EMF exposure ‘did not indicate strong or consistent associations with cancer’. Other exposures in the industry have been little considered. In 2010, cancer registration (incidence) data were incorporated into the UK cohort, and an analysis of cancer incidence for the period 1973–2008 was published in 2012 [13]. This report showed an occupational excess of mesothelioma but no excess of asbestos-induced lung cancer. There were also significant excesses of nasal cancer, and small intestine cancer in female employees; based on relatively small numbers of cases. An updated analysis of these UK data has been carried out aiming to provide a more complete monitoring of cancer risks in this cohort, and to identify any other types of cancer that merit further investigation. Details of exposure to electric and magnetic fields in this industry have been described before [5,14]. A long list of other occupational exposures present in parts of the industry before 1997 has also been published [15]. The cohort has been used in the past to test hypotheses [5–8], but this report is designed to generate rather than test occupational hypotheses.

## Methods

The study population and computerized data have been described previously [5–8,13]. The cohort comprises 83 284 employees (72 352 males and 10 932 females) of the former Central Electricity Generating Board (CEGB) of England and Wales. The earlier cohort [13] has been reduced in size because 639 employees who moved to Scotland had to be deleted from the study files because a Data Sharing Agreement (DSA) is no longer in place with the General Register Office (GRO) for Scotland. This small percentage change is not expected to result in an important selection bias. All employees had a minimum of 6 months of employment with some period of employment between 1973 and 1982. The total cohort was subdivided into three categories based on the work location (industry sector) of the first known job: power stations (*n* = 52 928), substation or transmission sites (*n* = 3359), and non-operational sites (*n* = 21 966). There were a further 3985 employees for whom no job history was available and 1046 employees whose work history could not be classified. These latter two were combined into a single ‘unclassifiable industry sector’ category.

NHS Digital (and its forerunners) supplied mortality and cancer registration follow-up particulars. NHS Digital is the national provider of information, data and IT systems for commissioners, analysts and clinicians in health and social care in England. By the study closing date (31 December 2015), 36 302 workers had died, 1090 workers had emigrated, 44 543 workers were traced alive and 1349 workers were untraced. After excluding the 1349 untraced workers and 319 workers whose deaths had been identified only by the former UK Department of Health and Social Security (DHSS) (and for whom cancer incidence data were not available), a total of 81 616 employees were considered for the cancer incidence analysis.

Cancer incidence in the cohort was compared with expected values based on incidence rates for England and Wales, taking sex, age and calendar period into account; calculations were carried out with the EPICURE programme [16], using the double precision DOS version 2.12 (2002) of DATAB. Study subjects were entered into the person-years-at-risk (pyr) after the first 6 months of employment or the date of computerization for the region of their employment, whichever was later. Individuals were removed from the pyr on the date of death, date of emigration or the end of 2015, whichever was the earlier. Study subjects did not contribute to observed or expected numbers after their 100th birthday, in case some subjects in later age groups had been traced alive incorrectly.

Standardized registration ratios (SRRs) by malignant neoplasm (MN) site were provided by the ratios of observed and expected numbers of cancer cases to a baseline of 100. *P*-values and 95% confidence intervals (CIs) were calculated on the basis that cancer occurs as a Poisson process, and all tests of statistical significance were two-tailed. More detailed analyses were also carried out by year of hire (1926–59, 1960–69, 1970–82), period from hire irrespective of how long any individual works in the industry (0–19, 20–29, 30–39, ≥40 years), period from leaving employment (still employed or left employment <10 years ago, left employment 10–19 years ago, left employment 20–29 years ago, left employment >30 years ago), duration of employment (<10 years (i.e. 0.5–9.9 years), 10–19 years, ≥20 years), industry sector of first known employment in the industry (power stations, transmission facilities, non-operational sites), and type of work (manager, engineer, administrative and clerical, industrial worker, building construction). For the first four variables, tests for trend (linear component) [17] were carried out (e.g. was there a tendency for SRRs to increase or decrease with year of hire). Tests for heterogeneity [17] were carried out for the last two variables (e.g. could the differences in SRRs by industry sector represent no more than random variation in sub-groups). These tests assumed a similar null hypothesis: no trend and homogeneous SRRs. All analyses only consider contemporaneous categories for the summation of pyr.

This study was established with the approval of the Central Ethical Committee of the British Medical Association, and the author is accredited by the Office for National Statistics as an ‘Approved Researcher’. The current protocol was approved by the University of Birmingham’ Science, Technology, Engineering and Mathematics Ethical Review Committee (project code ERN_13-0676). Computer analyses were carried out in accordance with the terms of an active Data Sharing Agreement (DSA) with NHS Digital. A privacy notice is available at http://www.emfs.info/research/studies/cegb-cohort/update/. One condition of the DSA was that findings based on fewer than five observed cases would not be published. The study has never contained any information on ethnicity, medical histories or lifestyle factors, and since 2013, no information on names, addresses, National Insurance or NHS numbers.

## Results

Table 1 shows site-specific MNs for male and female employees. Compared with national rates, all MNs combined were slightly (though highly significantly, *P* < 0.001) below expectation for males (Obs 19 223, SRR 97) and close to expectation for females (Obs 2149, SRR 101). In males, significant deficits are shown for MNs of the tongue, mouth, pharynx, oesophagus, rectum, liver, pancreas, larynx and lung. Significant excesses are shown for skin cancer (excluding melanoma) (Obs 5616, SRR 106, *P* < 0.001), mesothelioma (Obs 763, SRR 326, *P* < 0.001) and prostate cancer (Obs 4298, SRR 106, *P* < 0.001). In females, a significant deficit is shown for MN of the cervix, and significant excesses are shown for MN of the small intestine (Obs 13, SRR 220, *P* < 0.05), nasal cavities (Obs 11, SRR 407, *P* < 0.001) and breast (Obs 758, SRR 110, *P* < 0.01). Findings for MN of the brain and all leukaemia combined were unexceptional.

**Table 1.** Incidence of MNs in UK Electricity Generation and Transmission workers, 1973–2015 (71 185 males, 10 431 females)

Site of MN	ICD-10	Males				Females			
		Obs	Exp	SRR	95% CI	Obs	Exp	SRR	95% CI
Lip	C00	30	31.7	95	65–133	Sup.^a^			
Tongue	C01–02	75	108.7	69	55–86	11	7.6	145	76–252
Mouth	C03–06	74	113.1	65	52–82	10	8.8	114	58–203
Salivary gland	C07–08	41	40.7	101	73–135	Sup.^a^			
Pharynx	C09–14	104	163.8	63	52–77	Sup.^a^			
Oesophagus	C15	574	646.4	89	82–96	28	35.4	79	54–113
Stomach	C16	899	902.4	100	93–106	44	41.2	107	79–142
Small intestine	C17	61	59.7	102	79–130	13	5.9	220	123–367
Large intestine	C18	1587	1604.5	99	94–104	158	155.3	102	87–119
Rectum	C19–21	1052	1134.6	93	87–98	69	79.3	87	68–110
Liver	C22	185	242.9	76	66–88	13	15.9	82	45–136
Gallbladder	C23–24	98	90.0	109	89–132	16	12.2	131	78–208
Pancreas	C25	457	551.9	83	75–91	56	53.6	105	80–135
Other digestive	C26	32	39.0	82	57–114	Sup.^a^			
Nose and sinuses	C30–31	34	36.2	94	66–130	11	2.7	407	214–708
Larynx	C32	186	279.6	67	57–77	5	6.5	77	28–171
Lung and bronchus	C33,34	3162	3909.3	81	78–84	240	243.1	99	87–112
Bone	C40,41	22	26.1	84	54–126	Sup.^a^			
Melanoma	C43	444	446.0	100	91–109	69	67.4	102	80–129
Skin, other	C44	5616	5288.9	106	103–109	494	464.4	106	97–116
Mesothelioma	C45	763	234.0	326	304–350	Sup.^a^			
Connective tissue	C47,C49	98	96.3	102	83–124	10	9.3	108	55–192
Peritoneum	C48	21	20.4	103	65–155	7	5.3	132	58–261
Breast	C50	52	42.0	124	93–161	758	688.9	110	102–118
Cervix	C53	—	—			38	59.3	64	46–87
Uterus	C54	—	—			95	103.0	92	75–112
Ovary	C56	—	—			116	106.5	109	90–130
Prostate	C61	4298	4073.3	106	102–109	—	—		
Testis	C62	89	105.9	84	68–103	—	—		
Other genital	rem.^b^ C51–63	66	66.7	99	77–125	24	24.0	100	66–147
Kidney	C64	495	520.6	95	87–104	31	35.7	87	60–122
Bladder	C67	1257	1249.9	101	95–106	39	43.7	89	64–121
Other urinary	C65–66, C68	117	103.8	113	94–135	7	5.8	121	53–239
Eye	C69	34	34.7	98	69–135	5	3.7	135	50–300
Brain	C70–72	335	324.9	103	93–115	21	29.6	71	45–107
Thyroid	C73	48	49.5	97	72–128	11	16.3	67	35–117
Other endocrine glands	C74–75	12	14.6	82	45–140	Sup.^a^			
Secondary and unspecified cancers	C76–80	794	828.2	96	89–103	82	81.9	100	80–124
Hodgkin’s disease	C81	73	81.5	90	71–112	9	8.1	111	54–204
Non-Hodgkin’s lymphoma	C82–85	695	680.3	102	95–110	64	67.5	95	74–120
Multiple myeloma	C90	311	299.5	104	93–116	22	26.1	84	54–126
Leukaemia	C91–95	497	504.8	98	90–107	42	39.1	107	78–144
Acute lymphoid leukaemia	C91.0	12	15.8	76	41–129	Sup.^a^			
Chronic lymphoid leukaemia	C91.1	238	212.4	112	98–127	15	13.6	110	64–178
Acute myeloid leukaemia	C92.0, C92.5	134	156.0	86	72–101	19	14.6	130	81–200
Chronic myeloid leukaemia	C92.1	48	49.0	98	73–129	Sup.^a^			
Other leukaemia	rem.^b^ C91–95	65	71.7	91	71–115	5	5.0	100	37–222
All MNs	140–209^c^	19 223	19 822.0	97	96–98	2149	2122.9	101	97–106

^a^Sup = findings supressed because of confidentiality concerns about ‘disclosive’ data.

^b^rem = remainder.

^c^Excluding ‘skin, other’, ICD-10 C44.

Findings in Table 1 were examined to ascertain whether smoking-related cancers other than lung cancer were in deficit in male employees. For other cancer sites identified as capable of being caused by smoking cigarettes (oral cavity, pharynx, nasal cavity and paranasal sinuses, larynx, oesophagus, stomach, pancreas, liver, kidney, ureter, urinary bladder, myeloid leukaemia) [18], the SRR was significantly below expectation (Obs 4680, SRR 91, 95% CI 88–93). The SRR for cancers not judged to be capable of being caused by smoking (and excluding skin other than melanoma and unspecified neoplasms) was significantly elevated (Obs 10 587, SRR 107, 95% CI 105–109). However, the SRR for cancers not considered to be caused by smoking or asbestos exposures (further exclusion of mesothelioma) was not significantly elevated (SRR 101, 95% CI 99–104).

Findings from Table 1 were reviewed and all MNs, mesothelioma, MN of the skin (excluding melanoma) lung, breast and prostate in male workers, and MN of the small intestine, nasal cavities and breast in female workers were selected for further investigation.

Table 2 shows observed and expected numbers of cancer registrations for all MNs in male workers by year of hire, period from hire, period from leaving employment, duration of employment, industry sector and type of work. There was a highly significant positive trend with the period from leaving employment although this was dependent on a low SRR in a single category (still employed and left less than 10 years ago). There was also highly significant heterogeneity in the findings by industry sector and type of work; SRRs were lower in transmission and non-operational site workers compared with those in power station workers, and SRRs were lower in managers, engineers and clerical (including administrative) workers compared with those in industrial and construction workers.

**Table 2.** Incidence of all MNs combined (excluding skin other than melanoma) and MN of the skin (excluding melanoma) in 71 185 male UK Electricity Generation and Transmission workers, by year of hire, period from hire, period from leaving employment, duration of employment, industry sector and type of work, 1973–2015

	All MNs				MN of the skin			
	Obs	Exp	SRR	9 5% CI	Obs	Exp	SRR	95% CI
Year of hire								
1926–59	5850	6014.9	97	95–100	1668	1525.5	109	104–115
1960–69	7764	7957.0	98	95–100	2302	2123.0	108	104–113
1970–82	5609	5850.1	96	93–98	1646	1640.4	100	96–105
Test for trend			*P* = NS				*P* = *	
Period from hire (years)								
0–19	2006	2076.6	97	92–101	305	329.0	93	83–104
20–29	3514	3793.1	93	90–96	821	755.8	109	101–116
30–39	6011	6099.1	99	96–101	1576	1589.3	99	94–104
≥40	7692	7853.2	98	96–100	2914	2614.7	111	108–116
Test for trend			*P* = NS				*P* = **	
Period from leaving employment (years)								
<10^a^	4837	5373.1	90	88–93	872	898.0	97	91–104
10–19	6865	6859.1	100	98–103	1780	1615.3	110	105–115
20–29	5626	5686.7	99	96–102	2114	1949.7	108	104–113
≥30	1895	1903.2	100	95–104	850	825.8	103	96–110
Test for trend			*P* = ***				*P* = NS	
Duration of employment (years)								
<10	3564	3624.2	98	95–102	976	967.4	101	95–107
10–19	6616	6715.7	99	96–101	1887	1760.4	107	102–112
≥20	9043	9482.1	95	93–97	2753	2561.1	108	104–112
Test for trend			*P* = NS				*P* = NS	
Industry sector								
Power stations	13 745	13 570.1	101	100–103	3757	3599.5	104	101–108
Transmission	857	980.6	87	82–93	348	270.2	129	116–143
Non-operational	3508	4268.1	82	80–85	1302	1188.3	110	104–116
Unclassifiable^b^	1113	1003.1	111	105–118	209	230.9	91	79–103
Test for heterogeneity			*P* = ***				*P* = ***	
Type of work								
Managers	196	260.4	75	65–86	69	69.6	99	78–125
Engineers	4294	5092.0	84	82–87	1703	1449.5	118	112–123
Admin, clerical	1034	1232.4	84	79–89	358	325.8	110	99–122
Industrial	12 469	12 146.2	103	101–105	3266	3192.3	102	99–106
Building, constr.	250	217.0	115	102–130	52	54.6	95	72–124
Not known	980	873.9	112	105–119	168	197.1	85	73–99
Test for heterogeneity			*P* = ***				*P* = ***	
Total	19 223	19 822.0	97	96–98	5616	5288.9	106	103–109

NS, not significant.

^a^Includes still employed.

^b^Unclassifiable work history or no work history.

*P < 0.05, **P < 0.01, ***P < 0.001,

Table 2 shows findings for MN of the skin (excluding melanoma) in male workers. A significant negative trend is shown with year of hire (SRRs tend to be lower with more recent hires), and a significant positive trend is shown with a period from hire. There was also highly significant heterogeneity in the findings by industry sector and type of work; SRRs were higher in transmission workers compared with those in power station and non-operational site workers and SRRs were higher in engineers and clerical workers compared with those in industrial and construction workers.

Table 3 shows findings for mesothelioma and MN of the lung in males; for mesothelioma, there was a highly significant negative trend with year of hire and a highly significant positive trend with period from hire (SRRs were higher with later periods from hire). There was a highly significant positive trend with duration of employment. There was also highly significant heterogeneity in the findings by industry sector and type of work; SRRs were higher in power station workers compared with those in transmission and non-operational site workers, and SRRs were lower in clerical workers compared with those in all other types of work. Very different patterns are shown for MN of the lung. There was a highly significant positive trend with year of hire (SRRs were higher with more recent decades of hire), and a highly significant negative trend with period from hire (SRRs were lower with later periods from hire). There was a highly significant negative trend with duration of employment. There was also highly significant heterogeneity in the findings by industry sector and type of work; SRRs were higher in power station workers compared with those in transmission and non-operational site workers, and SRRs were lower in managers, engineers and clerical (including administrative) workers compared with those in industrial and construction workers.

**Table 3.** Incidence of mesothelioma and MN of the lung in 71 185 male UK Electricity Generation and Transmission workers, by year of hire, period from hire, period from leaving employment, duration of employment, industry sector and type of work, 1973–2015

	Mesothelioma				MN of the lung			
	Obs	Exp	SRR	95% CI	Obs	Exp	SRR	95% CI
Year of hire								
1926–59	311	65.9	472	422–527	999	1330.8	75	71–80
1960–69	334	97.1	344	309–382	1297	1587.4	82	77–86
1970–82	118	71.0	166	138–198	866	991.1	87	82–93
Test for trend			*P* = ***				*P* = ***	
Period from hire (years)								
0–19	29	14.7	197	135–280	500	548.4	91	83–99
20–29	72	38.5	187	147–234	741	847.0	87	81–94
30–39	221	72.6	304	266–347	942	1142.2	82	77–88
≥40	441	108.2	408	371–447	979	1371.7	81	67–76
Test for trend			*P* = ***				*P* = ***	
Period from leaving employment (years)								
<10^a^	125	44.6	280	234–333	1125	1420.5	79	75–84
10–19	282	80.6	350	311–393	1042	1300.8	80	75–85
20–29	267	80.2	333	295–375	739	904.8	82	76–88
≥30	89	28.5	312	252–383	256	283.2	90	80–102
Test for trend			*P* = NS				*P* = NS	
Duration of employment (years)								
<10	70	41.3	170	133–213	644	658.3	98	90–106
10–19	228	77.3	295	259–335	1191	1336.1	89	84–94
≥20	465	115.4	403	368–441	1327	1914.9	69	66–73
Test for trend			*P* = ***				*P* = ***	
Industry sector								
Power stations	616	160.9	383	354–414	2416	2690.2	90	86–93
Transmission	20	12.2	164	103–249	119	190.5	62	52–74
Non-operational	92	52.0	177	143–216	384	803.1	48	43–53
Unclassifiable^b^	35	8.9	393	278–541	243	225.4	108	95–122
Test for heterogeneity			*P* = ***				*P* = ***	
Type of work								
Managers	10	2.9	345	175–615	17	56.2	30	18–47
Engineers	209	66.2	316	275–361	381	940.2	41	37–45
Admin, clerical	7	13.5	52	23–103	138	245.0	56	48–56
Industrial	486	141.3	344	314–376	2356	2421.8	97	93–101
Building, constr.	19	2.4	792	491–1213	45	46.1	98	72–130
Not known	32	7.6	421	293–587	225	199.9	113	99–128
Test for heterogeneity			*P* = ***				*P* = ***	
Total	763	234.0	326	304–350	3162	3909.3	81	78–84

NS, not significant.

^a^Includes still employed.

^b^Unclassifiable work history or no work history.

***P < 0.001.

Table 4 shows findings for MN of the prostate. There was a significant positive trend with duration of employment. There was also highly significant heterogeneity in the findings by type of work; SRRs were higher in engineers and clerical workers compared with those in other types of work.

**Table 4.** Incidence of MN of the prostate in 71 185 male UK Electricity Generation and Transmission workers, by year of hire, period from hire, period from leaving employment, duration of employment, industry sector and type of work, 1973–2015

	MN of the prostate			
	Obs	Exp	SRR	95% CI
Year of hire				
1926–59	1254	1167.5	107	102–114
1960–69	1789	1660.6	108	103–113
1970–82	1255	1245.2	101	95–107
Test for trend			*P* = NS	
Period from hire (years)				
0–19	153	143.4	107	91–125
20–29	548	565.4	97	89–105
30–39	1448	1356.9	107	101–112
≥40	2149	2007.6	107	103–112
Test for trend			*P* = NS	
Period from leaving employment (years)				
<10^a^	516	503.3	103	94–112
10–19	1719	1540.7	112	106–117
20–29	1560	1510.7	103	98–109
≥30	503	518.6	97	89–106
Test for trend			*P* = NS	
Duration of employment (years)				
<10	703	724.7	97	90–104
10–19	1417	1354.4	105	99–110
≥20	2178	1994.2	109	105–114
Test for trend			*P* = **	
Industry sector				
Power stations	2887	2779.4	104	100–108
Transmission	213	207.1	103	90–117
Non-operational	1002	899.9	111	105–118
Unclassifiable^b^	196	186.8	105	91–120
Test for heterogeneity			*P* = NS	
Type of work				
Managers	47	52.1	90	67–119
Engineers	1300	1108.9	117	111–124
Admin, clerical	278	246.8	113	100–127
Industrial	2454	2459.5	100	96–104
Building, constr.	47	42.9	110	81–144
Not known	172	163.1	106	91–122
Test for heterogeneity			*P* = ***	
Total	4298	4073.3	106	102–109

NS, not significant.

^a^Includes still employed.

^b^Unclassifiable work history or no work history.

**P < 0.01, ***P < 0.001.

Corresponding findings for MN of the small intestine and the nasal cavities in female workers were also calculated. Both findings were based on small numbers and are not tabulated because of concerns about ‘disclosive’ data. There were no significant trends or heterogeneity in the findings.

Table 5 shows findings for MN of the breast for male and female workers. There was significant heterogeneity in the findings for females by type of work; SRRs were higher in clerical workers and workers with unknown job type compared with those found in engineers and industrial workers.

**Table 5.** Incidence of MN of the breast in 71 185 male and 10 431 female UK Electricity Generation and Transmission workers, by year of hire, period from hire, period from leaving employment, duration of employment, industry sector and type of work, 1973–2015

	Males				Females			
	Obs	Exp	SRR	95% CI	Obs	Exp	SRR	95% CI
Year of hire								
1926–59	18	12.5	144	88–223	51	38.3	133	100–174
1960–69	19	16.8	113	70–173	145	128.5	113	96–132
1970–82	15	12.8	117	68–189	562	522.1	108	99–117
Test for trend			*P* = NS				*P* = NS	
Period from hire (years)								
0–19	6	4.7	128	52–266	168	152.7	110	94–128
20–29	11	8.2	134	71–233	225	209.0	108	94–122
30–39	13	12.9	101	56–168	267	241.0	111	98–125
≥40	22	16.2	136	87–202	98	86.2	114	93–138
Test for trend			*P* = NS				*P* = NS	
Period from leaving employment (years)								
<10^a^	16	11.8	136	80–216	179	164.7	109	94–126
10–19	13	14.0	93	52–155	228	194.1	118	103–134
20–29	16	12.3	130	77–207	213	214.7	99	87–113
≥30	7	3.9	180	79–355	138	115.5	120	101–141
Test for trend			*P* = NS				*P* = NS	
Duration of employment (years)								
<10	10	7.9	127	64–226	428	400.9	107	97–117
10–19	16	14.3	112	66–178	240	211.0	114	100–129
≥20	26	19.8	131	88–190	90	77.0	117	95–143
Test for trend			*P* = NS				*P* = NS	
Industry sector								
Power stations	33	28.7	115	80–160	273	253.6	108	95–121
Transmission	2	2.1	95	16–315	11	12.5	88	46–153
Non-operational	16	9.1	176	104–279	417	374.0	112	101–123
Unclassifiable^b^	Sup.^c^				57	48.8	117	89–150
Test for heterogeneity			*P* = NS				*P* = NS	
Type of work								
Managers	Sup.^c^				Sup.^c^			
Engineers	18	10.8	167	102–258	13	13.2	98	55–164
Admin, clerical	5	2.6	192	70–426	566	496.0	114	105–124
Industrial	28	25.7	109	74–155	130	140.0	93	78–110
Building, constr.	Sup.^c^				Sup.^c^			
Not known	Sup.^c^				47	38.4	122	91–161
Test for heterogeneity			*P* = NS				*P* = *	
Total	52	42.0	124	93–161	758	688.9	110	102–118

NS, not significant.

^a^Includes still employed.

^b^Unclassifiable work history or no work history.

^c^Sup = findings suppressed because of confidentiality concerns about ‘disclosive’ data.

*P < 0.05.

## Discussion

Overall, this study showed a clear occupational excess of mesothelioma with no matching excess of lung cancer, and unexceptional findings for brain tumours and leukaemia. Strengths of the study include its size and length of follow-up with a correspondingly large number of cancer cases, including rare cancers. Limitations include the absence of smoking data, other lifestyle data and detailed pre-1973 work histories. The latter meant that first known job had to be used to categorize individuals by industry sector and type of work (55% of the cohort had some employment within the industry before personnel records were computerized). Some misclassification in the sub-group analyses will have occurred although only 2% of power station workers had later recorded periods of working in the transmission sector and 6% of transmission workers had later recorded periods of working in power stations.

Incidence of all MNs combined was below expected in males, and morbidity from lung cancer was markedly below expectation. Findings for other smoking-related cancers were consistent with the hypothesis that this skilled workforce had below average smoking habits; a low prevalence of smoking in this industry has been published previously [13].

Mesothelioma was significantly elevated for males in all industry sectors and in all types of work except administration and clerical work; with little sign of the effects of asbestos risk having played itself out (2006–2010: Obs 159, SRR 317, 95% CI 271–369; 2011–2015: Obs 169, SRR 298, 95%CI 255–345). The most likely explanation for the excess of mesothelioma in transmission and non-operational site workers is occasional or earlier (pre-1973) periods of working at power stations where asbestos was used to lag pipes and boilers [13]. It is estimated that in the UK in 2004 there were 1937 mesotheliomas and 2223 lung cancer cases caused by earlier asbestos exposure [19]. This estimate considers that there will be 1.1 lung cancers for every mesothelioma caused by asbestos. This 1.1:1 ratio does not seem to be applicable to the UK electricity supply industry workers. The numbers in this study are so large that the absence of a lung cancer excess related to asbestos is very unlikely to be a chance finding. Low smoking prevalence in the cohort explains this to some extent, but there must be other unrecognized factors in operation.

The excess of small intestine cancer in female employees with no comparable excess in male employees indicates that occupational exposures are not important in this excess, as it is difficult to imagine exposures in this industry that would be unique to female workers. There were no important contrasts in the more detailed analyses for this excess. Risk factors for small intestine cancer have been little studied, but there is some evidence that ‘risk factors are similar to those seen with colon cancer (meat intake) and stomach cancer (salt-cured and smoked foods)’ [20]. As findings for MN of the large intestine and stomach in females were close to expectation in this study, they do not offer any indirect support for a dietary explanation for the excess MN of the small intestine, and confident interpretation of the excess is not possible.

There was a marked excess of nasal cancer in female employees with no matching excess in male employees. Nasal cancer, particularly adenocarcinoma, is associated with some occupational exposures, including hard wood dust, leather dust and hexavalent chromium exposure [21]. Exposures to leather dust and hexavalent chromium are not present in the industry under study, although there are carpentry shops in power stations. However, none of the female cases ever worked in such shops and bystander exposure seemed an unlikely explanation for this excess when there was no corresponding excess in male workers. There were no important contrasts in the detailed analyses that were carried out for this excess, and it seems unlikely that this excess is due to occupational exposures in this industry. Nevertheless, it remains possible that some of the nasal cancers in females are occupational in origin due to unrecognized factors in this industry or unknown employment in other industries.

Exposure to sunlight is an accepted risk factor for skin cancer (non-melanoma), and the higher risk in transmission workers could be attributable to outdoor working. Unfortunately, individual data on occupational and non-occupational sun exposure are not available for study, but general information on dress habits and outdoor working in the industry could be examined in future studies in terms of skin cancer fourth digit codes (e.g. wearing of short-sleeved shirts and skin cancer of the upper limb ICD-10 C44.6).

Several studies of workers potentially exposed to EMF have reported increased risks of male breast cancer [22–25], although three cohort studies of electric utility workers reported no overall excess [2–4]. There were no important contrasts in more detailed analyses that were carried out for male breast cancer in this study. The excess of breast cancer in female workers was based, mainly on an excess in administrative and clerical workers; making occupational exposures involvement in this excess unlikely.

There was a significant trend for prostate cancer in relation to duration of employment. The reason for this is unclear, but it is possible that sedentary working is involved [26]. Examination of this hypothesis would involve collection of additional work history data, possibly as part of a nested case–control study.

In conclusion, the overall elevated incidence found for mesothelioma almost certainly reflects the late health effects of earlier incidental asbestos exposure in this industry. This report highlights the need for further research in a number of areas, including the conditions that lead to some asbestos-exposed cohorts not having clear excesses of asbestos-induced lung cancer. It would be useful to review incident cancers of the nasal cavities and the small intestine in female workers in the electricity supply industry in other countries. Nested case–control studies could also be usefully carried out on skin cancer and prostate cancer. Such studies would require collection of additional data, but permissions are currently not in place to obtain such data.

These findings have implications for clinicians and policymakers; they reinforce the importance of regulations that protect workers from asbestos exposure and the advice given to outdoor workers concerning sun exposure. They provide indirect evidence that further control of magnetic fields exposure is probably not needed, and indicate that employees who have been exposed to asbestos should continue to be encouraged not to smoke to reduce the risk of asbestos-related lung cancer.

## Funding

The work and the costs of an open access publication were supported by a research award from the electricity supply industry and funding was administered by the Energy Networks Association (ENA).

## References

[1] Thériault G, Goldberg M, Miller AB et al. Cancer risks associated with occupational exposure to magnetic fields among electric utility workers in Ontario and Quebec, Canada, and France: 1970–1989. Am J Epidemiol 1994;139:550–572. 8172168 10.1093/oxfordjournals.aje.a117046

[2] Savitz DA, Loomis DP Magnetic field exposure in relation to leukemia and brain cancer mortality among electric utility workers. Am J Epidemiol 1995;141:123–134. 7817968 10.1093/oxfordjournals.aje.a117400

[3] Kelsh MA, Sahl JD Mortality among a cohort of electric utility workers, 1960–1991. Am J Ind Med 1997;31:534–544. 9099354 10.1002/(sici)1097-0274(199705)31:5<534::aid-ajim6>3.0.co;2-t

[4] Johansen C, Olsen JH Risk of cancer among Danish utility workers—a nationwide cohort study. Am J Epidemiol 1998;147:548–555. 9521181 10.1093/oxfordjournals.aje.a009486

[5] Harrington JM, Nichols L, Sorahan T, van Tongeren M Leukaemia mortality in relation to magnetic field exposure: findings from a study of United Kingdom electricity generation and transmission workers, 1973–97. Occup Environ Med 2001;58:307–314. 11303079 10.1136/oem.58.5.307 PMC1740133

[6] Sorahan T, Nichols L, van Tongeren M, Harrington JM Occupational exposure to magnetic fields relative to mortality from brain tumours: updated and revised findings from a study of United Kingdom electricity generation and transmission workers, 1973–97. Occup Environ Med 2001;58:626–630. 11555682 10.1136/oem.58.10.626 PMC1740052

[7] Sorahan T, Nichols L Mortality from cardiovascular disease in relation to magnetic field exposure: findings from a study of UK electricity generation and transmission workers, 1973–1997. Am J Ind Med 2004;45:93–102. 14691973 10.1002/ajim.10314

[8] Sorahan T, Kheifets L Mortality from Alzheimer’s, motor neuron and Parkinson’s disease in relation to magnetic field exposure: findings from the study of UK electricity generation and transmission workers, 1973–2004. Occup Environ Med 2007;64:820–826. 17626136 10.1136/oem.2006.031559 PMC2095390

[9] Santibáñez M, Bolumar F, García AM Occupational risk factors in Alzheimer’s disease: a review assessing the quality of published epidemiological studies. Occup Environ Med 2007;64:723–732. 17525096 10.1136/oem.2006.028209 PMC2078415

[10] National Radiological Protection Board. ELF Electromagnetic Fields and the Risk of Cancer: Report of an Advisory Group on Non-ionising Radiation. Chilton, Oxfordshire: NRPB, 2001.

[11] International Agency for Research on Cancer. IARC Monographs on the Evaluation of Carcinogenic Risks to Humans. Non-ionising Radiation, Part 1: Static and Extremely Low Frequency (ELF) Electric and Magnetic Fields, vol. 80 Lyon: IARC Press, 2002. PMC5098132 12071196

[12] Kheifets L, Bowman JD, Checkoway H et al. Future needs of occupational epidemiology of extremely low frequency electric and magnetic fields: review and recommendations. Occup Environ Med 2009;66:72–80. 18805878 10.1136/oem.2007.037994

[13] Sorahan T Cancer incidence in UK electricity generation and transmission workers, 1973–2008. Occup Med (Lond) 2012;62:496–505. 22949586 10.1093/occmed/kqs152

[14] Renew DC, Cook RF, Ball MC A method for assessing occupational exposure to power-frequency magnetic fields for electricity generation and transmission workers. J Radiol Prot 2003;23:279–303. 14582720 10.1088/0952-4746/23/3/305

[15] Harrington JM, McBride DI, Sorahan T, Paddle GM, van Tongeren M Occupational exposure to magnetic fields in relation to mortality from brain cancer among electricity generation and transmission workers. Occup Environ Med 1997;54:7–13. 9072027 10.1136/oem.54.1.7 PMC1128628

[16] Preston DL, Lubin JH, Pierce DA, McConney ME. Epicure Users Guide. Seattle, USA: Hirosoft International Corp, 1993.

[17] Breslow NE, Day NE. Statistical Methods in Cancer Research. Volume II—The Design and Analysis of Cohort Studies. IARC Scientific Publication no. 82. Lyon: IARC Press, 1987. 3329634

[18] International Agency for Research on Cancer. IARC Monographs on the Evaluation of Carcinogenic Risks to Humans. Tobacco Smoke and Involuntary Smoking, vol. 83 Lyon: IARC Press, 2004. PMC4781536 15285078

[19] Rushton L, Hutchings SJ, Fortunato L et al. Occupational cancer burden in Great Britain. Br J Cancer 2012;107(Suppl 1):S3–S7. 22710676 10.1038/bjc.2012.112 PMC3384015

[20] Chow WH, Linet MS, McLaughlin JK, Hsing AW, Chien HT, Blot WJ Risk factors for small intestine cancer. Cancer Causes Control 1993;4:163–169. 8481495 10.1007/BF00053158

[21] International Agency for Research on Cancer. IARC Monographs on the Evaluation of Carcinogenic Risks to Humans. A Review of Human Carcinogens: Arsenic, Metals, Fibres, and Dusts, vol. 100c. Lyon: IARC Press, 2011.

[22] Tynes T, Andersen A, Langmark F Incidence of cancer in Norwegian workers potentially exposed to electromagnetic fields. Am J Epidemiol 1992;136:81–88. 1415133 10.1093/oxfordjournals.aje.a116423

[23] Demers PA, Thomas DB, Rosenblatt KA et al. Occupational exposure to electromagnetic fields and breast cancer in men. Am J Epidemiol 1991;134:340–347. 1877594 10.1093/oxfordjournals.aje.a116095

[24] Matanoski GM, Breysse PN, Elliott EA Electromagnetic field exposure and male breast cancer. Lancet 1991;337:737. 1672204 10.1016/0140-6736(91)90325-j

[25] Pollán M, Gustavsson P, Floderus B Breast cancer, occupation, and exposure to electromagnetic fields among Swedish men. Am J Ind Med 2001;39:276–285. 11241560 10.1002/1097-0274(200103)39:3<276::aid-ajim1015>3.0.co;2-b

[26] Shephard RJ Physical activity and prostate cancer: an updated review. Sports Med 2017;47:1055–1073. 27844337 10.1007/s40279-016-0648-0

